# Direct Anterior vs. Direct Lateral Approach Total Hip Arthroplasty for Displaced Femoral Neck Fracture

**DOI:** 10.3390/jcm12155019

**Published:** 2023-07-30

**Authors:** Ron Ben Elyahu, Nissim Ohana, Eltaieb Agabaria, Ahmad Biadsi, David Segal, Eyal Yaacobi, Ezequiel Palmanovich, Michael Markushevich, Yaron Shraga Brin

**Affiliations:** Department of Orthopedic Surgery, Meir Medical Center, Tel-Aviv University, Kfar-Saba 4428164, Israel; ronitaly@gmail.com (R.B.E.); niso62@yahoo.com (N.O.); tayeeb.helo@gmail.com (E.A.); ahmadbiadsi@gmail.com (A.B.); dudisegal@gmail.com (D.S.); yaacobi.eyal@gmail.com (E.Y.); ezepalm@gmail.com (E.P.); michaelmark01@gmail.com (M.M.)

**Keywords:** hip fracture, total hip arthroplasty, direct lateral approach, direct anterior approach, clinical outcomes

## Abstract

Background: This study compared outcomes of the direct anterior approach (DAA) and direct lateral approach (DLA) for treating displaced femoral neck fractures in active elderly patients. Methods: This retrospective study included active elderly patients who sustained a displaced femoral neck fracture and underwent a cementless total hip arthroplasty either with a supine DAA or a decubitus DLA. Patients were assessed using the Harris hip score at discharge and at a 6-week follow-up. Results: A total of 41 women and 18 men were included in the study. Of those, 22 underwent DLA and 37 received DAA, all performed by the same team. In both groups, 69% were women, mean age was 70 years, and mean BMI was 25.2. Mean hemoglobin loss was 2.3 g/dl between admission and the first post-operative day in both groups. Similar numbers in each cohort were discharged home rather than to a rehabilitation center. The patients who underwent the DAA experienced a 2-day reduction in their hospital stay compared to the DLA group (4.2 ± 1.9 vs. 6.8 ± 3.7, respectively; *p* < 0.001). The Harris hip score in the DAA group was significantly higher at the 6-week follow-up than in the DLA group (87.23 ± 7.75 vs. 81.23 ± 7.67, respectively; *p* < 0.031). Conclusions: The patients who underwent THA with the DAA demonstrated better short term outcomes compared to the alternative approach for displaced femoral neck fractures in active elderly patients. DAA helped patients regain independence faster and might decrease hospitalization and rehabilitation costs. Based on these results, we recommend using the DAA for active elderly patients with a displaced femoral neck fracture.

## 1. Introduction

Hip fractures are an international public health problem, and there are approximately 1.5 million hip fractures worldwide per year. The number of fractures continues to rise as the population ages, and it is estimated that the number of hip fractures worldwide will increase to 2.6 million by 2025 and 4.5 million in the 2050’s [1]. Despite a decline in the incidence of hip fractures for both men and women, the overall number of hip fractures continues to rise due to the aging population. This persistent upward trend remains a significant public health concern. As the population ages, it becomes increasingly crucial to address this issue with preventive measures and effective interventions to mitigate the impact of hip fractures on individuals and healthcare systems [1]. Nearly half (45–50%) of hip fractures occur in the subcapital (femoral neck) region, of which the majority are displaced [2]. Surgical intervention remains the main treatment for displaced femoral neck fractures, and it seems that the main challenge is the post-operative rehabilitation, including gaining back independence, walking abilities, and physical function. Different surgical options for displaced femoral neck fractures are either hemiarthroplasty or total hip arthroplasty. In both cases, the primary goal of treatment is to return patients to their pre-fracture level of function. Unfortunately, among patients who were living independently prior to a hip fracture, only about half can walk unaided after the injury [3,4]. Although hemiarthroplasty is a very common operative treatment for this injury, recently there has been an increased interest in total hip arthroplasty (THA) for this injury, with superior clinical results in the long-term [5,6,7]. There are three main surgical approaches for THA:

The postero-lateral approach—the surgeon typically detaches or splits the gluteus maximus, piriformis, and external rotators to gain access to the hip joint capsule.

The direct lateral approach—the surgeon divides the abductor mechanism to gain access to the joint capsule. (Detailed explanation of this approach can be found in the Methods section of this manuscript).

The direct anterior approach—the surgeon makes an incision on the anterior aspect of the hip, allowing direct access to the hip joint between certain muscles and soft tissues without cutting or splitting those muscles. (Detailed explanation of this approach can be found in the Methods section of this manuscript).

In our department we use either the direct lateral approach (DLA) or the direct anterior approach (DAA) for THA.

The aim of this study was to assess the short term outcomes of THA for displaced femoral neck fracture in both the DLA and the DAA.

## 2. Methods

### 2.1. Patients

Active patients aged 59–85 who sustained a displaced femoral neck fracture and were treated by THA were included in the study. Patients were divided into two cohorts according to the surgical approach: DLA or DAA.

In 2020, we implemented a significant change in our surgical approach for hip fractures, transitioning from the direct lateral approach to the direct anterior approach. After achieving a learning curve with the new approach, we conducted a comparative evaluation of 37 consecutive patients that were operated on using the DAA, with the previous 22 patients having been operated on with the DLA.

All patients were operated on within 48 h following arrival at the hospital.

### 2.2. Surgical Approach

#### 2.2.1. Direct Anterior Approach

Patients were positioned in a supine position on a regular operating table that could be flexed at the hip. We used a modified Hueter approach to the hip joint. We started with a transverse skin incision on the anterior aspect of the thigh. The incision started 5 cm distal to the anterior superior iliac spine and progressed up to 10 cm laterally on the anterior aspect of the thigh. The DA approach involved exposing the tensor fascia lata and dividing its perimysium. The interval between the sartorius and tensor fascia lata was used. The lateral head or reflected portion of the rectus was retracted medially. Anterior capsulectomy was performed and the fractured femoral neck was exposed. Osteotomy of the femoral neck was performed, and the femoral head and neck were extracted using a corkscrew. The acetabulum and the femoral canal were then prepared in a routine fashion. Exposure of the femoral canal involved selected soft tissue releases on the posterior aspect of the femoral neck while preserving the abductor mechanism and the short external rotators.

#### 2.2.2. Direct Lateral Approach

Patients were positioned in a lateral decubitus position on a regular operating table. The DLA was performed by placing the incision over the greater trochanter and dividing the underlying fascia lata. The abductor mechanism was divided up to its middle third, retracting the anterior half anteriorly. A capsulotomy was performed, and the femoral neck fracture was exposed. A femoral neck osteotomy was performed, and the femoral head and neck were removed. The acetabulum and femoral canal were prepared in the conventional manner.

### 2.3. Implants

All patients underwent THA using the Corail cementless stem and Pinnacle cementless cup (Depuy Synthes, Warsaw, IN, USA) with the same surgical team.

### 2.4. Measurements

The Harris hip score (HHS) was used to evaluate pain, support, limp, walking distance, and total HHS [7]). The patients were evaluated at discharge by a physical therapist and at a 6-week follow-up in the outpatient clinic by one of the authors.

We also assessed duration of hospital stay and percentage of patients in each cohort who were discharged home from the department.

### 2.5. Data Analysis

Data were described as mean ± standard deviation (SD) for continuous variables and as numbers and percentages for nominal variables. These were analyzed using chi-square or Fisher’s exact test and *t*-test or Mann–Whitney test for metric data, according to the distribution of the variables. *p*-values < 0.05 were considered statistically significant. The data were analyzed using SPSS-25 software package (IBM Corp., Armonk, NY, USA).

## 3. Results

A total of 59 patients, aged 59–83 years, were included in the study. Among them, 37 underwent THA via the DAA and 22 via the DLA. Age, sex, BMI, and comorbidities were similar in both groups. Demographics and comorbidities are summarized in Table 1.

All patients were operated on within 48 h from admission to the hospital. Hemoglobin loss during the procedure was similar in both groups.

### 3.1. Functional Outcomes upon Discharge

Some of the functional outcomes assessed using the Harris hip score had already improved upon discharge in the DAA group, including support while walking and walking distance. All other measurements, including the total HHS were similar in both groups at that stage.

Patients in the DAA arm had a significantly shorter duration of hospital stay following the operation compared to the DLA arm (4.2 ± 1.9 vs. 6.8 ± 3.7 days; *p* = 0.001). The results are summarized in Table 2.

### 3.2. Functional Outcomes at 6-Week Follow-Up

Pain was similar in both groups. Patients in the DAA group reported better walking abilities. They needed less support while walking and limped less than patients in the DLA group. Most importantly, they reported that they could walk for longer distances. Patients in the DAA group had significantly better total HHS. Functional results at discharge and at the 6-week follow-up are summarized in Table 3.

## 4. Discussion

The objective of this study is to evaluate the very early outcomes of patients who underwent THA for a displaced femoral neck fracture.

THA is a treatment for the long run in active patients suffering from a displaced femoral neck fracture. Usually, the clinical results of this procedure are evaluated several years following the operation. In this study, we decided to conduct an early evaluation of patients undergoing THA for a displaced femoral neck fracture, in order to assess which of the two surgical approaches we used contributes to a better recovery and a shorter hospitalization. We believe that the early outcomes are very important especially in active elderly patient, since the aim of the treatment for a displaced femoral neck fracture is an early ambulation in order to avoid complications such as deep-vein thrombosis, pulmonary embolism, and nosocomial infections.

We found better short-term functional outcomes for patients who were operated on using the DAA (Table 3).

Total Harris hip scores were similar in both groups at discharge. Yet, there were some advantages for the patients in the DAA arm, which already appeared in the first days after surgery.

### 4.1. Pain

It has already been described by others that the DAA causes less pain compared to other approaches, mainly since it is an inter-muscular approach, and there is less damage to the soft tissue surrounding the joint. Bergin and co-workers reported lower concentrations of serum creatine kinase in patients that were operated on using the DAA compared to the posterior approach. Creatine kinase represents muscle damage, which should be lower in the DAA since it is an intermuscular approach. They also showed lower levels of inflammatory mediators in the DAA, especially the TNF-a [7]. Several other studies found that patients undergoing THA for hip osteoarthritis with a DAA had less pain [8,9,10,11,12,13]. It could be attributed to the smaller skin incision used in the minimally invasive DAA, the lesser innervation of the excised joint capsule in the anterosuperior area, or to the blunt preparation with minimal muscle damage. Surprisingly, in this study we found that patients in both cohorts had similar pain scores upon discharge from the hospital and at the 6-week follow-up. We believe that these similar pain scores reflect the fact that our patients sustained acute pain due to the femoral neck fracture. The similar pain scores are attributed mainly to the acute trauma of the femoral head and surrounding soft tissues. Hence, it is difficult to find a significant difference between the two approaches.

### 4.2. Function

Many studies found better functional results among patients undergoing THA in the DAA, as compared to the DLA in patients with hip osteoarthritis [10,11,12,13,14,15,16]. Similar to those studies, but this time following a displaced femoral neck fracture, we showed better functional results in the DAA group at the 6-week follow-up. We even showed that patients in the DAA cohort had better walking abilities already upon discharge, but the total HHS score was similar in both groups at that very early stage and improved significantly by the 6-week follow-up.

### 4.3. Duration of Surgery

Duration of surgery involves a learning curve, especially when changing from a well-established approach to a new one. Other writers approached the issue of the duration of surgery. Alecci and coauthors [10] and Mjaaland and associates [15] showed slightly shorter operation times for the DLA compared to the DAA. On the other hand, other authors found equal surgery times for the two procedures [12,13,14,17]. Our results showed a trend of shorter operative time for the DAA compared to the DLA, although they were not statistically significant. With a larger cohort, they might have become statistically significant. We think that with the continuation of the learning curve, the DAA may become a significantly faster procedure compared to the DLA, and a future study may prove this. Shorter procedures may contribute to a reduced rate of post-operative infections, a reduction of blood loss, and saving operative time.

### 4.4. Bleeding

Several researchers reported more blood loss in DLA patients compared to DAA patients [12,15,16,18]. In our study, we found equal rates of blood loss in both procedures. We assume that the main blood loss in our cohorts was a consequence of the displaced femoral neck fracture and not the surgical approach. Hence, we found no significant difference in the rate of blood loss for the two cohorts.

### 4.5. Length of Stay

The DAA cohort had shorter hospital stays than the DLA group. Short hospital stays are the policy of most healthcare systems worldwide, to avoid nosocomial infections and other in-hospital complications, as well as saving resources. It has been shown that hip osteoarthritis patients who were operated on using the DAA had shorter hospital stays compared to patients who were operated on using the DLA [10,11,17,19]. When treating active elderly patients who sustain a displaced femoral neck fracture, short hospital stays are even more important because those patients are usually more affected by other comorbidities and are at a higher risk of in-hospital complications. In addition, short hospitalization and fast rehabilitation may contribute to avoiding psychological complications. Hence, treatment objectives would also include early mobilization with early home discharge, in order to avoid complications.

### 4.6. Summation

It is already known and discussed in other studies in the literature that THA carried out using the DAA gives better results than THA that is carried out using the DLA. The DAA has advantages over the DLA in the following aspects: less pain in the DAA, better functional outcomes in the short term, less bleeding with the DAA, and shorter hospital stays with DAA. All these parameters are well known and studied for patients who suffer from osteoarthritis of the hip joint. Our study evaluated the same parameters, but this time concentrated on patients who suffered from a femoral neck fracture. Our study suggests that the better results for the DAA also stand for patients who sustained a femoral neck fracture. Our study has some limitations: it is a retrospective study and it includes a small numbers of patients. We believe that this issue should be studied further using prospective studies with a greater number of patients.

## 5. Conclusions

This study shows that a total hip arthroplasty for a displaced femoral neck fracture in an active elderly patient performed by the direct anterior approach provides better early functional outcomes. In addition, patients who were operated on using the direct anterior approach were able to return home earlier than their counterparts who were operated on using the direct lateral approach.

The objective of this study was to evaluate the very early outcomes of those two groups. With the ever-increasing demand for total hip arthroplasty for this injury, and limited resources, there is a real necessity to provide a high level of care and early return to full function. This could be achieved using the direct anterior approach.

## Figures and Tables

**Table 1:** Characteristics of patients in each surgical group.

Characteristic	Direct Lateral (N = 22)	Direct Anterior (N = 37)	*p*-Value
Female	67.7%	72.7%	0.677
Age	70.86 ± 4.51	69.7 ± 5.53	0.86
BMI	25.4 ± 3.8	25.6 ± 3.3	0.837
IHD	8.1%	9.1%	1
CVA	0%	4.5%	0.373
End stage renal disease	0%	4.5%	0.373
Diabetes mellitus	21.6%	18.2%	1
COPD	2.7%	9.1%	0.549
Psychiatric disorder	5.4%	4.5%	1
Malignancy	13.5%	9.1%	0.702

**Table 2:** Surgical outcomes in each group.

Outcome	Direct Lateral (N = 22)	Direct Anterior (N = 37)	*p*-Value
Operated within 48 h	100%	100%	1
Duration of surgery, minutes; mean ± SD	105 ± 15.5	97 ± 15.7	0.065
Hemoglobin prior to surgery, mean ± SD	12.99 ± 1.23	13.06 ± 1.54	
Hemoglobin loss	2.282 ± 1	2.3 ± 0.95	0.92
Post-operative hospital stay, days; mean ± SD	6.778 ± 3.74	4.162 ± 1.92	0.001
Discharge to rehab center,	11 (50%)	14 (37.8%)	0.361
Dislocations during a 6-week follow-up	0	0	1

**Table 3:** Functional results at discharge and at 6-week follow-up.

Assessment Period	Variable	Direct Lateral	Direct Anterior	*p*-Value
At discharge	Pain	26.82 ± 8.94	28.65 ± 5.85	0.345
	Support	1.7 ± 0.7	2.0 ± 0	0.021
	Limp	2.00 ± 0	1.89 ± 0.46	0.275
	Walking distance	6.91 ± 1.47	7.68 ± 0.94	0.018
	Stairs	0	3.0 ± 0.52	0.01
	Total HHS	47.15 ± 9.94	49.62 ± 6.65	0.242
At 6-week follow-up	Pain	42.73 ± 1.90	42.38 ± 1.99	0.5
	Support	5.41 ± 2.46	7.38 ± 3.04	0.014
	Limp	6.91 ± 2.37	8.32 ± 2.21	0.024
	Walking distance	7.73 ± 1.83	9.62 ± 1.51	0.000
	Stairs	2.05 ± 0.95	2 ± 0.94	0.859
	Total HHS	81.23 ± 7.87	87.23 ± 7.75	0.006

Scores are given according to the Harris hip score [6].

## Data Availability

Not applicable.
